# The Moderating Role of Willpower as a Personality Trait in the Relationship Between Social Influence and Moral Disengagement Contradiction

**DOI:** 10.1002/brb3.70506

**Published:** 2025-05-05

**Authors:** Nesrullah Okan

**Affiliations:** ^1^ Department of Educational Sciences, Guidance and Psychological Counseling Fırat University Elazığ Turkey

**Keywords:** attitudes towards immigrants, moral contradiction, moral disengagement, personality, social influence, willpower

## Abstract

**Objective:**

The objective of this study is to examine the moderating role of willpower, conceptualized as a personality trait, in the relationship between social influence and moral disengagement towards migrants. The mediating role of spiritual contradiction is also investigated to understand the interplay between individual traits, moral contradictions, and external social pressures.

**Method:**

Data were collected from 720 participants using validated self‐report measures. Structural equation modeling (SEM) was employed to test the proposed relationships among social influence, moral disengagement, spiritual contradiction, and willpower. Moderation and mediation analyses were conducted to evaluate the hypothesized model.

**Results:**

The findings reveal that social influence significantly predicts moral disengagement. This relationship is partially mediated by spiritual contradiction, which amplifies disengagement by reflecting tensions between internal moral values and external norms. In addition, the study found that willpower moderates this relationship by reducing the negative impact of social influence on moral disengagement. Individuals with higher levels of willpower demonstrate greater resistance to moral disengagement and maintain moral consistency despite external pressures and moral contradictions.

**Conclusions:**

This study underlines the pivotal function of personality traits and spiritual dimensions in shaping moral processes. The findings have practical applications for ethical education and interventions designed to enhance moral resilience in varied social contexts.

## Graphical abstract

The graphical abstract provides a visual representation of the intricate interplay among willpower, social influence, spiritual contradiction, and moral disengagement. The model posits that social influence has the potential to enhance moral disengagement; however, this effect is tempered by willpower and mediated by spiritual contradiction. Individuals who possess higher levels of willpower demonstrate augmented cognitive control, thereby diminishing the influence of social influence on moral disengagement. Conversely, individuals with lower willpower exhibit a heightened vulnerability to social pressure, which, through spiritual contradiction, amplifies their susceptibility to moral disengagement. This theoretical framework contributes to our understanding of the role of executive function and self‐regulation in ethical decision‐making, emphasizing the neurocognitive mechanisms underlying moral disengagement in social contexts.

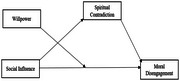

## 1 Introduction

### 1.1 Definition of the Problem

Attitudes towards migrants and refugees are shaped by complex psychological, moral, and social processes, with social influence playing a particularly significant role (Cialdini and Goldstein 2004). Social influence mechanisms, including normative pressures, conformity, and social modeling, have been shown to shape individuals' thoughts, attitudes, and behaviors in ways that align with dominant social norms. Within the context of migration, these mechanisms can drive attitudes ranging from prosocial to exclusionary, depending on the prevailing social climate and normative cues (Esses et al. 2013).For instance, exposure to inclusive discourse and contact with individuals who hold positive attitudes toward migrants can promote empathy, while environments characterized by hostility or fear can reinforce negative biases and moral disengagement. However, while the impact of social influence is significant, it is not the sole factor in shaping attitudes toward migrants. A related psychological mechanism that plays a role in this process is moral disengagement, which refers to the cognitive process by which individuals justify or rationalize harmful behaviors toward others by disengaging from moral self‐regulation (Bandura 1999). In the context of anti‐immigrant attitudes, social influence can facilitate this process by legitimizing discriminatory behaviors through collective narratives or group norms, thereby rendering exclusionary practices seemingly moral. Furthermore, the notion of moral ambivalence assumes a pivotal role in this process, encapsulating the inherent discord that emerges when individuals' personal values find themselves in conflict with prevailing social norms (Hill and Pargament 2003). Within the context of migration, individuals may concur with the ethical imperative to assist those in need while concurrently being subjected to social pressures that encourage exclusionary attitudes. This interplay between social influence, moral disengagement, and moral ambivalence underscores the intricacy of the formation and perpetuation of attitudes toward migrants. Furthermore, the concept of willpower, defined as an individual's capacity to resist social influences, emerges as a pivotal moderating factor in this process. Willpower, as a mechanism of self‐regulation, enables individuals to maintain alignment with their core values and moral principles despite external pressures (Baumeister et al. 2007; Okan 2019). It is hypothesized that individuals with strong willpower are less susceptible to conforming to socially reinforced discriminatory attitudes and are more likely to uphold ethical commitments towards migrants and refugees. The present study aims to examine the interplay between social influence, moral disengagement, and moral ambivalence, while also exploring the moderating role of willpower in shaping attitudes towards migrants. By integrating perspectives from social psychology, ethics, and spirituality, this research provides a multidimensional framework for understanding how individuals navigate the complex moral landscape of migration attitudes. The findings are expected to offer insights into how social influence mechanisms can either reinforce exclusionary biases or promote ethical, compassionate responses towards migrant populations.

### 1.2 Literature Background

In the field of social psychology, the shaping of individuals' behaviors in the face of social norms and influences has been the subject of research for many years. Social influence refers to the tendency of individuals to adapt to pressures or norms from other people in their environment, and this can also affect individuals' moral decisions (Cialdini and Goldstein 2004; Ekşi et al. 2021). Social influence theories explain how individuals respond to norms, rules, and expectations in their social environment. For example, Kelman's (1958) social influence model, which includes the stages of “adaptation, identification, and internalization”, sheds light on how individuals internalize social pressures from their environment and how these pressures are reflected in individuals' attitudes. In addition, Asch's (1951) conformity experiments showed that individuals can change even a response they know to be correct under group pressure. These findings emphasize that social influences can play a strong role in moral decisions.

In particular, the theory of “moral disengagement” developed by Albert Bandura (1999) provides an important framework explaining the processes by which individuals rationalize their behaviors contrary to ethical principles. This theory reveals the mechanisms by which individuals can legitimize their negative behaviors by deviating from ethical norms under pressures triggered by social influences. Bandura identified eight main mechanisms of moral disengagement and explained how individuals rationalize their negative behavior: moral justification, allocation of responsibility, minimization of consequences, and dehumanization (Bandura 1999). For example, an individual may justify discriminatory attitudes towards immigrants in order to “preserve social order” and this is an example of the moral disengagement process.

In this context, it appears that social influences may increase individuals' tendencies to develop moral detachment. Especially attitudes towards vulnerable groups such as immigrants may be shaped by such processes. In addition to Bandura, Tajfel and Turner's (1979) social identity theory explains how individuals' attitudes towards ingroup and outgroup members are formed and how these processes can trigger moral disengagement mechanisms. Migrants can be subjected to dehumanization processes through stereotypes and prejudices, often by being placed in an outgroup category (Haslam 2006).

Spiritual contradiction refers to the contradiction between individuals' spiritual values and environmental pressures, and how this contradiction affects individuals' moral and psychological processes has gained importance in recent years (Hill and Pargament 2003). Spiritual contradictions can trigger moral disengagement processes by weakening the inner harmony of individuals. Pargament et al. (2011) emphasized the negative effects of spiritual contradictions on individuals' capacity to cope with stress and revealed the importance of this contradiction in individuals' decision‐making mechanisms.

The capacity of individuals to resist such social and moral pressures is related to the concept of willpower. Willpower refers to the self‐regulation capacity of individuals and plays a decisive role in both psychological and social processes (Baumeister et al. 2007). In the study conducted by APA (2012), which investigated the causes of stress for three years, lack of willpower was ranked first among the sources of stress. The problem of willpower lies at the root of many problems, from struggling with weight to regular exercise; from waking up on time to saving money. This is an indication of how important willpower is for people. Is willpower an illusion, as Nahmias (2014) and Harris (2012) say; is willpower the reality itself, as List (2019) says; or is willpower nothing more than the directional program of a computer made of flesh, as Coyne (2015) says? These statements actually contain clues about the structure of the will that has been discussed so far. First, the idea that free will is an illusion or an illusion reflects the discussion over about forty years (Lavazza 2016; Yu 2012). This is based on studies in the field of neuroscience (Libet 1985; Libet et al. 1983). In a way, determinism has been tried to be verified with neuroscience studies. It has been claimed that the brain is activated for preference before the person makes a choice and thus makes the person prefer that situation (Haynes 2011; Soon et al. 2008). According to this idea, the choice has been realized in the brain before the person makes his/her choice and there is no possibility for the person to make another choice. Therefore, free will is nothing but an illusion (Crick 1994; Greene and Cohen 2004; Cashmore 2010; Harris 2012). However, Kane (2011) stated that the fact that determinism is true cannot prevent the existence of free will. Along with these studies, some recent studies have evaluated that the changes occurring in the brain nerves may be the reflection of many different factors (Schurger et al. 2012). This change reduces the gap between the determinist perspective and free will (Roskies 2012). Bode et al. (2014) stated that what is overlooked in the studies is the person's experiences, behavior patterns, and intentions. Therefore, the brain is likely to be affected by habits and behavior patterns. Haggard (2008) stated that seeing unconscious brain activities as responsible for our conscious behaviors would eliminate free will and cause great confusion. Therefore, he stated that the main point to be emphasized is the power of the will. Baumeister et al. (1998) emphasized that willpower increases the capacity of individuals to achieve their long‐term goals and make ethical decisions. In addition, Tangney et al. (2004) showed that individuals with low willpower are more open to social pressures and this may facilitate moral disengagement processes.

### 1.3 Originality and Contributions of the Study

Although research examining the impact of social influence on individuals' moral judgments has generated extensive literature, more comprehensive models to understand the complex dynamics of these interactions are limited. In particular, studies focusing on the relationship between social influence and moral disengagement lack models that include mediating and moderating variables. In this context, the framework that this study presents by combining social influence with variables such as moral ambivalence and voluntariness fills an important gap in the literature.

Spiritual contradiction refers to the contradiction between individuals' intrinsic values and external social norms, and the impact of this concept on moral disengagement has been addressed in a limited number of studies (Hill and Pargament 2003; Pargament et al. 2011; Okan and Okan 2024). There is no comprehensive examination of how moral ambivalence plays a mediating role in social influence processes. While most studies have focused on the effects of spiritual contradiction on individuals' general psychological adjustment (Hill et al. 2000; Okan and Ören 2021), the elaboration of these effects in the context of moral disengagement has been neglected in the literature.

However, the regulatory role of willpower in moral processes shaped by social influences has not been sufficiently addressed in the literature. Willpower is a critical self‐regulatory capacity that enables individuals to maintain internal control mechanisms in the face of environmental influences (Baumeister et al. 1998; Tangney et al. 2004). However, there are limited studies on how willpower influences the relationship between social influence and moral disengagement. The inclusion of such a moderating variable in the model may contribute to understanding the protective mechanisms that reduce individuals' moral deviance.

The unique contribution of this study is that it extends the effect of social influence on moral disengagement with variables representing individuals' values and self‐regulation processes, such as moral ambivalence and willpower. Existing models in the literature generally focus on the direct relationship between social influence and moral processes and ignore the complex psychological mechanisms underlying these processes (Cialdini and Goldstein 2004; Bandura 1999). Therefore, this study offers a new perspective on understanding individuals' moral disengagement processes by addressing social influence theories from a more comprehensive perspective.

### 1.4 Purpose of the Study and Hypotheses

This study aims to understand the effects of social influence on moral disengagement towards refugees and to examine the mediating role of moral ambivalence and the moderating effect of willpower in this process. Social influence is an important psychological concept in terms of understanding how individuals shape their behaviors and attitudes in the face of social norms and pressures. Moral disconnect refers to the tendency of individuals to rationalize certain behaviors by deviating from ethical and moral principles. In this context, moral contradiction represents the contradiction between individuals' moral values and social norms and is considered a critical mediating variable that can affect moral disengagement processes. Will, on the other hand, can play a regulatory role in this process by expressing individuals' self‐regulation capacities in the face of environmental influences and internal contradictions.

The research aims to provide a new model for understanding the relationships between social influence, moral ambivalence, moral disengagement, and willpower, and to provide an in‐depth analysis of how these dynamics shape individuals' social attitudes. Furthermore, this model is expected to make theoretical and practical contributions to the understanding of attitudes towards vulnerable groups such as migrants and to promote social cohesion.

**H1**: Social influence has a significant effect on moral detachment towards refugees.

- This hypothesis suggests that social influence may increase individuals' tendency to deviate from ethical norms and develop negative attitudes toward refugees. Social pressures and norms can strongly influence individuals' moral decision‐making processes (Cialdini and Goldstein 2004).

**H2**: Spiritual contradiction mediates the relationship between social influence and moral disengagement.

- Moral contradiction refers to the contradiction that individuals experience between social norms and their spiritual values, and this contradiction is thought to play an important role in individuals' processes of developing moral disengagement. Moral contradiction may be a critical mechanism shaping the impact of social influence on moral decisions (Hill and Pargament 2003).

**H3**: Will regulates the relationship between spiritual contradiction and moral disengagement.

- Willpower represents individuals' capacity for internal control and can influence the strength of the relationship between moral ambivalence and moral disengagement. A strong willpower may facilitate individuals to remain faithful to their ethical values in the face of social influences (Baumeister et al. 1998). This hypothesis suggests that willpower, as a regulating factor, may increase individuals' resilience in spiritual and moral processes.

The research model is delineated in Figure 1.

**FIGURE 1:**
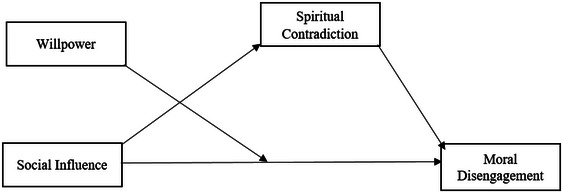
Research model.

## 2 Method

### 2.1 Research Design

This study employs a cross‐sectional research design, utilizing quantitative methods to investigate the relationships among key variables. Cross‐sectional designs are particularly effective for examining the associations between individual traits and behavioral outcomes by collecting data from a sample at a specific point in time (Levin 2006; Setia 2016). The study focuses on the influence of social factors, spiritual contradiction, and willpower— conceptualized as a personality trait—on moral disengagement towards refugees. The research was conducted among university students in Turkey, and the findings are interpreted within the framework of personality psychology, emphasizing individual differences and their role in moral and social processes.

In the study, structural equation modeling (SEM) was used to test the direct, indirect, and conditional effects between variables. SEM is an effective analysis method for testing complex relationships between multiple variables and examining non‐linear paths (Kline 2016; Byrne 2016). In this study, the use of SEM within Andrew Hayes' Model 5 framework made it possible to understand how social influence influences moral disengagement towards refugees through moral ambivalence (mediating variable) and willpower (moderating variable).

SEM analyses were conducted with AMOS and SPSS PROCESS plug‐ins, and the recommended procedures for model validation and testing processes were followed (Hair et al. 2019). The reliability and validity of the variables were evaluated by model fit criteria and bootstrap analyses (Schumacker and Lomax 2016; Hayes 2022). While the limitations of the cross‐sectional design include the inability to finalize causal inferences, the strengths of SEM partially compensated for these limitations. In this context, by providing an innovative perspective in social psychology, important findings on how social influence shapes individual moral decision processes through willpower and moral ambivalence have been revealed.

### 2.2 Sample

As demonstrated in Table 1, an analysis of the demographic and subjective evaluation information of the participants revealed a balanced gender distribution, with 50.4% of subjects identifying as female and 49.6% as male. This distribution is significant in terms of the generalizability of the results. In the distribution of the participants according to the grade level, it was determined that 1st‐year students constituted the largest group with 46.0%, followed by 2nd year (21.7%), 3rd year (14.7%), 4th year (13.1%), and other (4.6%) categories. This situation shows that the research is especially focused on undergraduate students. When the academic achievement perceptions of the participants are analyzed, it is seen that the majority of them evaluate their achievement level as “moderate” (63.3%), followed by those who describe it as “good” (33.9%) and “poor” (2.8%). In the evaluation of socioeconomic status, most of the participants (73.6%) described their situation as “moderate”, while 13.9% described it as “bad” and 12.5% as “good”. In the study, the sample size of 720 people increases the power of statistical analyses and shows that it can make important contributions in the context of social psychology.

**TABLE 1:** Participants' demographic and subjective evaluation information table.

Variable	Option	Frequency (*n*)	Percentage (%)	Valid percentage (%)	Cumulative percentage (%)
**Gender**	**Woman**	363	50.4	50.4	50.4
	**Male**	357	49.6	49.6	100.0
**Class level**	**Grade 1**	331	46.0	46.0	46.0
	**Grade 2**	156	21.7	21.7	67.6
	**Grade 3**	106	14.7	14.7	82.4
	**Grade 4**	94	13.1	13.1	95.4
	**Other**	33	4.6	4.6	100.0
**Academic success**	**Bad**	20	2.8	2.8	2.8
	**Center**	456	63.3	63.3	66.1
	**Good**	244	33.9	33.9	100.0
**Socio‐economic status**	**Bad**	100	13.9	13.9	13.9
	**Center**	530	73.6	73.6	87.5
	**Good**	90	12.5	12.5	100.0
	**Total**	**720**	**100.0**	**100.0**	**100.0**

### 2.3 Measurement Tools

In this study, four different scales were used to measure the levels of social influence, moral contradiction, moral disengagement towards refugees, and willpower. Detailed explanations of these scales are given below:

**Social influence scale** (Ekşi et al. 2021)

The social influence scale was developed to measure the social influence levels of individuals. This self‐report, seven‐point Likert‐type scale consists of 13 items and has a single‐factor structure. The total variance of the scale was 46.637% and Cronbach's alpha reliability coefficient was found to be 0.941. The results of item total‐return analyses and confirmatory factor analysis (χ^2^/sd = 2.786; RMSEA = 0.076; SRMR = 0.09; CFI = 0.929; GFI = 0.915) revealed that the scale is a valid and reliable measurement tool. This scale is used to measure the level of social influence of university students.

**Spiritual contradiction scale** (Okan et al. 2024)

The spiritual contradiction scale was developed to measure the inconsistencies between individuals' spiritual beliefs, values, and actions. The scale consists of 12 items and two sub‐dimensions (internal contradiction and external contradiction). The factor analyses showed that the scale explained 64% of the total variance. The Cronbach's alpha reliability coefficient of the scale was 0.943, and in the confirmatory factor analysis (χ^2^/df = 1.682; RMSEA = 0.066; SRMR = 0.010; CFI = 0.952; GFI = 0.890), goodness of fit values were found to be at an acceptable level. The spiritual contradiction scale is an effective tool for determining the spiritual inconsistencies of individuals.

**Moral disengagement scale for refugees** (Okan et al. 2023)

The moral disengagement scale was developed to measure individuals' level of moral disengagement towards refugees. The scale consists of 28 items and five sub‐dimensions. The total variance explained was 64.251% and Cronbach's alpha reliability coefficient was calculated as 0.943. Confirmatory factor analysis results (χ^2^/df = 1.682; RMSEA = 0.066; SRMR = 0.010; IFI = 0.953; CFI = 0.952; GFI = 0.890) supported the structural validity of the scale. This scale is a valid and reliable instrument for assessing moral disengagement towards refugees.

**Willpower heptet scale** (Okan 2019)

The willpower heptet scale was developed to measure individuals' sub‐dimensions related to willpower such as decision‐making, self‐control, consistency, and responsibility. The scale consists of 38 items and 7 sub‐dimensions. Confirmatory factor analysis results (χ^2^/sd = 2.037; RMSEA = 0.049; SRMR = 0.08; CFI = 0.939; NFI = 0.901) show that the scale is a good fit. All item loadings were above 0.45 and the scale was proved to be valid and reliable at an acceptable level.

### 2.4 Data Collection Process

Data were collected through both online and face‐to‐face survey administration. While the online administration provided a wider geographical reach to the participants, the face‐to‐face administrations allowed the researcher to closely observe the individual participation process and guide the participants immediately with possible questions. The online surveys were delivered via a web platform that was easily accessible to the participants, while the face‐to‐face surveys were conducted on university campuses and other social settings deemed appropriate for the research. Participants were given detailed information about the process and voluntary participation was taken as a basis. Ethical rules were given great importance in the data collection process. The research was subjected to an ethical approval procedure committed to protecting the privacy and confidentiality of the participants. Written or electronic consent was obtained from the participants and it was stated that the questionnaire responses were made completely anonymous. All data were stored securely to be used only for the purposes of this research and were not shared with third parties. These procedures increased participant trust and ensured that the research was conducted in accordance with ethical standards.

### 2.5 Methods of Analysis

**Descriptive Analyses**: The data analysis process started with descriptive analyses including the validity and reliability tests of the scales used. In this context, Cronbach's Alpha reliability coefficient was calculated for each scale and it was determined that the internal consistency of the scales was at an acceptable level. In addition, factor analyses (exploratory and confirmatory) were used to examine the construct validity of the scales. The results of the analyses showed that the measurement tools used provided an appropriate and reliable structure for data collection.

**Correlation Analyses**: Pearson correlation analyses were applied to determine the main relationships between the variables. These analyses revealed the direction and power relations between the variables of social influence, moral contradiction, will, and moral disengagement towards refugees. The correlation values obtained showed the existence of significant relationships between the variables and that these relationships can be shaped in line with the hypotheses.

**Structural Equation Modelling**: SEM was used to test the mediating and moderating effects, which is the main objective of the study. SEM was chosen as an ideal method to test indirect and direct effects between variables. The model examined the effect of social influence on moral disengagement in the context of the mediating role of spiritual contradiction and the moderating effect of willpower. The goodness of fit values (such as RMSEA, CFI, and TLI) were evaluated to confirm that the overall fit of the model was acceptable. This analysis showed that the relationships between the variables were compatible with the theoretical framework and the model was sufficient to answer the research questions.

## 3 Findings

### 3.1 Descriptive Statistics

The descriptive statistics presented in Table 2 are of paramount importance for the analysis of the distribution and trends of respondents on the measured variables.

**TABLE 2:** Descriptive statistics.

Variable	(Mean)	(Std. Dev.)	Minimum	Maximum	(Skewness)	(Kurtosis)
**Willpower**	195.23	35.38	38.00	276.00	−0.501	1.372
**Social influence**	40.30	9.91	13.00	87.00	−0.498	0.168
**Moral disengagement**	69.96	10.60	27.00	90.00	−1.264	1.926
**Spiritual contradiction**	40.59	9.34	12.00	56.00	−0.606	0.408

**Willpower**: The mean score is 195.23 and there is a wide distribution for this variable (standard deviation = 35.38). The minimum value was 38 and the maximum value was 276. The negative skewness (−0.501) indicates that the levels of willpower are more concentrated towards higher values, while the positive kurtosis (1.372) indicates that the distribution is more pointed. This indicates that the participants generally exhibit strong willpower, but there are significant differences between them.

**Social Influence**: The mean score is 40.30 and the standard deviation is 9.91, and it is seen that this variable exhibits a narrower distribution. The minimum value is 13 and the maximum value is 87. The skewness value (−0.498) shows that the social influence scores tend to have high values, while the kurtosis value (0.168) shows that the distribution is within the limits of normality. This result suggests that the participants are moderately affected by social influence, but some individuals are more affected by this influence.

**Moral Disengagement**: The mean score is 69.96, indicating that the participant's level of moral disengagement is generally high. The standard deviation is 10.60, indicating a moderate variation. The skewness value (−1.264) shows that the scores are concentrated towards higher values, while the kurtosis value (1.926) shows the sharpness of the distribution. This indicates that the majority of the participants experience moral disengagement, but some individuals have lower values in this regard.

**Spiritual Contradiction**: With a mean score of 40.59 and a standard deviation of 9.34, it is understood that this variable shows a moderate level of difference among the participants. The minimum value is 12 and the maximum value is 56, and the negative skewness (−0.606) indicates that the scores are more concentrated on high values. The kurtosis value (0.408) indicates a distribution within the limits of normality. Spiritual contradiction levels show that the incompatibilities between the beliefs and behaviors of individuals are experienced at a moderate level.

As demonstrated in Table 3, the findings of the correlation analysis indicate a substantial and negative association between the willpower variable and moral disengagement (−0.330), spiritual contradiction (−0.560), and social influence (−0.658). These results imply that perceived moral disengagement, spiritual contradiction, and stress may intensify in the presence of diminished willpower. The moral disconnect variable exhibits significant positive relationships with spiritual contradiction (0.405) and social influence (0.313). This suggests that perceived moral disengagement may increase with spiritual contradictions and stress effect. Similarly, the positive relationship between spiritual contradiction and social influence (0.347) indicates that these two variables act in parallel. These results reveal important relationships between individuals' willpower and spiritual and psychological variables.

**TABLE 3:** Correlation Table.

Variables	Willpower	Moral disengagement	Spiritual contradiction	Social influence
**Willpower**	1.000	−0.330^*^	−0.560^*^	−0.658^*^
**Moral disengagement**	−0.330^*^	1.000	0.405^*^	0.313^*^
**Spiritual contradiction**	−0.560^*^	0.405^*^	1.000	0.347^*^
**Social influence**	−0.658^*^	0.313^*^	0.347^*^	1.000

^*^ *p* < 0.001.

### 3.2 Hypothesis Tests

As illustrated in Table 4, this model explores the impact of social influence, spiritual contradiction, and willpower on moral disengagement. It also discloses the substantial outcomes of the interactions between the variables. The explanatory level of the model was found to be *R* ^2^ = 0.284, and 28.4% of the variance in the moral disengagement variable was explained by the independent variables (*F* = 70.91, *p* < 0.001). The effect of social influence on moral disengagement is negative and significant (*B* = −1.22, *p* < 0.001), indicating that negative social influence increases the tendency of moral disengagement in individuals. Spiritual contradiction showed an increasing effect on moral disengagement (*B* = 0.28, *p* < 0.001), indicating that individuals' inner contradictions may cause disengagement in their moral attitudes. Willpower has a negative and significant effect on moral disengagement (*B* = −0.31, *p* < 0.001), indicating that strong willpower assumes a protective function against social influences and moral contradictions. Moreover, the significant interaction between social influence and willpower (*B* = 0.007, *p* < 0.001) indicates that willpower balances the damaging effects of social influence and reduces the negative effects on moral disengagement. This model makes an important contribution to understanding the dynamics of factors that influence individuals' moral judgments.

**TABLE 4:** Model summary related to social influence, spiritual contradiction, and will.

Variables	B (Beta)	Standard error (SE)	*t* value	*p* value	Lower limit (LLCI)	Upper limit (ULCI)
**Constant**	117.18	8.93	13.13	< 0.001	99.66	134.70
**Social influence (X)**	−1.22	0.16	−7.67	< 0.001	−1.53	−0.91
**Spiritual contradiction (M)**	0.28	0.04	6.34	< 0.001	0.19	0.37
**Willpower (W)**	−0.31	0.04	−8.74	< 0.001	−0.39	−0.24
**Social influence × Willpower (X×W)**	0.007	0.001	9.27	< 0.001	0.0054	0.0082

As demonstrated in Table 5, spiritual contradiction exerts a substantial mediating effect on the relationship between social influence and moral disengagement. As illustrated in Table 5, the indirect effect of social influence on moral disengagement is mediated through spiritual contradiction, and this effect is statistically significant (*B* = 0.092; BootSE = 0.021; BootLLCI = 0.051; BootULCI = 0.134). Since the bootstrap confidence intervals of the indirect effect do not contain zero, this relationship is statistically strongly supported. These findings suggest that social influence creates spiritual contradictions in individuals and these contradictions increase their moral disengagement levels. In other words, part of the total effect of social influence on moral disengagement is realized indirectly through spiritual contradiction. This situation reveals that negative social factors are not only limited to direct effects but also lead to the dissolution of moral values by increasing the moral contradictions of individuals.

**TABLE 5:** Indirect effects (mediating role of spiritual contradiction).

Mediating variable (*M*)	Impact (B)	BootSE	BootLLCI	BootULCI
Spiritual Contradiction (*M*)	0.092	0.021	0.051	0.134

**FIGURE 2:**
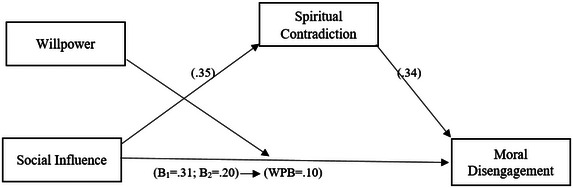
Research model results.

As illustrated in Figure 2, the study's findings offer a comprehensive explanation of the impact of social influence on the phenomenon of moral disengagement towards refugees. The analysis further elucidates the mediating role of moral ambivalence and the moderating effect of willpower in this context. First, the direct relationship between social influence and moral disengagement was found to be positive and significant (*B*1 = 0.31). This suggests that social norms and environmental influences may cause individuals to deviate from their moral values and exhibit more moral detachment toward refugees. Social influence has come to the fore as an important variable that paves the way for individuals to deviate from ethical standards due to external pressures.

When spiritual contradiction was included in the model as a mediating variable, the relationship between social influence and moral disengagement decreased (*B*2 = 0.20) and it was observed that spiritual contradiction played a partial mediating role in this relationship. Spiritual contradiction refers to the contradiction between individuals' internal values and external social norms, and it has been found that this contradiction may deepen moral disengagement processes. The positive and significant effect of moral ambivalence (*B* = 0.35) emphasizes the potential of social influence to create moral ambivalence in individuals and its negative effects on moral disengagement.

One of the most striking findings of the study is the moderating role of willpower (Willpower) in the relationship between social influence and moral disengagement. With the inclusion of willpower in the model, the effect of social influence on moral disengagement weakened further and decreased to WPB = 0.10. This finding indicates that individuals' level of willpower is a protective factor against the damaging effects of social influence. Individuals with high levels of willpower can protect their moral values by becoming more resistant to social norms. This shows that willpower is a critical psychological mechanism that strengthens the moral consistency of individuals.

In conclusion, the research findings explain how the relationship between social influence and moral disengagement is shaped by variables such as spiritual ambivalence and willpower. Spiritual ambivalence partially mediates the effect of social influence on moral disengagement, while willpower provides a mechanism that limits these effects and helps individuals maintain their moral consistency. The results of the study emphasize the importance of spiritual and willpower factors for individuals to develop resistance to social norms and strengthen their moral processes.

As demonstrated in Graph 1, the impact of social influence and varying levels of willpower on moral disengagement is illustrated. The graph shows that social influence increases moral disengagement at all levels of willpower, but this effect differs depending on the level of willpower. At low willpower levels, the effect of social influence on moral disengagement is the strongest, which is observed by the steeper slope compared to other levels. At the medium willpower level, the effect of social influence decreases, while at the high willpower level, this effect is weakest. The fact that the line is more horizontal indicates that individuals with high willpower are less affected by social influence and have lower levels of moral disengagement. As a result, as individuals' capacity for self‐control increases, the effect of social influence on moral disengagement decreases. This suggests that willpower can play a protective role against social influence and support individuals' moral consistency.

**FIGURE 1:**
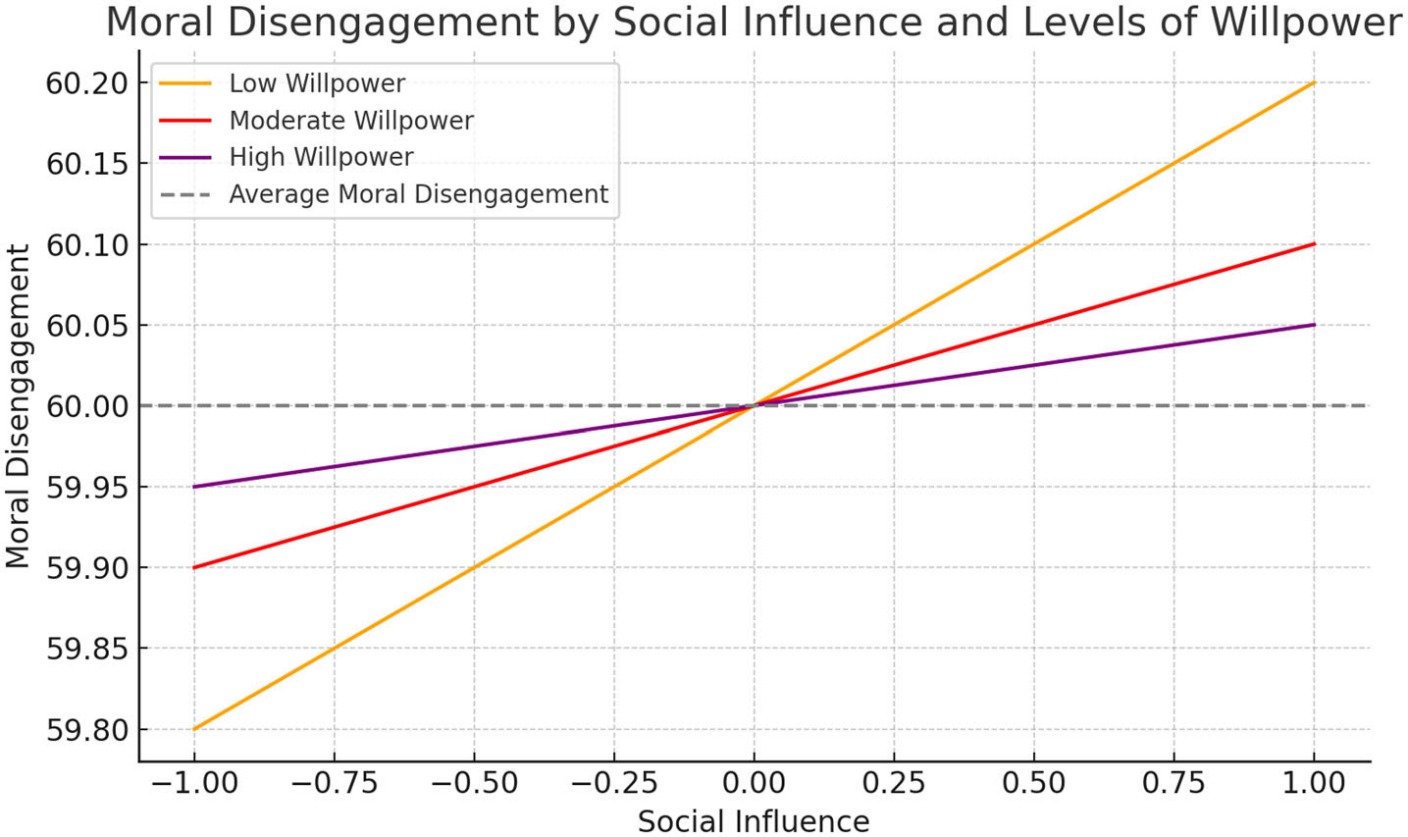
Moderator effect graph.

## 4 Conclusion, Discussion, and Suggestions

### 4.1 Results and Main Findings

This study revealed the impact of social influence on moral detachment towards refugees, the critical mediating role of moral ambivalence in this process, and the strong moderating factor of willpower. The findings of the study show that social influence increases the tendency of individuals to develop moral detachment towards refugees, but strong willpower plays a stabilizing role in this negative effect. Social influence stands out as an important factor that causes individuals to deviate from their ethical values towards refugees due to their tendency to adapt to environmental pressures. However, willpower helped individuals to resist the damaging consequences of these social influences.

At the same time, moral ambivalence has been identified as an important mechanism that deepens individuals' tendency to deviate from ethical norms towards refugees in this process. Moral ambivalence reflects the mismatch between individuals' internal values and beliefs and external social norms. The study showed that moral ambivalence partially mediates the effect of social influence on moral disengagement towards refugees and that these ambivalences may lead to greater deviations in individuals' ethical values. This suggests that individuals' coping with moral ambivalence may play a critical role in maintaining their ethical consistency towards refugees.

Willpower stood out as one of the most striking moderating factors among the findings of the study. Individuals with high levels of willpower were found to be more resistant to the negative consequences of social influences and had lower levels of moral detachment towards refugees. Willpower functions as a basic psychological mechanism that limits individuals' tendency to adapt to social pressures and maintains their ethical consistency towards refugees. It was found that the effect of social influence on moral detachment towards refugees was more pronounced at low levels of willpower, but this effect decreased significantly with increasing levels of willpower. This finding clearly shows that willpower plays a role in balancing the damaging effects of social influence and supporting individuals' ethical consistency.

Therefore, this study examined the direct and indirect effects of social influence on moral disengagement towards refugees and revealed the mediating role of moral contradiction in this process and the regulatory effect of willpower. The findings provide an important contribution to the social psychology literature and emphasize the importance of willpower development and strategies for coping with moral contradictions in order for individuals to maintain their ethical values towards refugees. These results provide an in‐depth understanding in terms of both theoretical frameworks and applied approaches.

### 4.2 Comparison of Findings With Literature

The findings of this study are in significant agreement with social psychological theories and previous literature. *Social exchange theory* suggests that individuals make cost and benefit evaluations in social relationships and that these processes may affect their moral and spiritual processes (Blau 1964). According to this theory, individuals may tend to adapt to the pressures and norms coming from their social environment, which may lead them to deviate from their ethical values. In our study, the finding that social influence has an increasing effect on moral detachment towards refugees supports this theory. Individuals' tendency to adapt to external social pressures may cause them to move away from ethical norms and develop a higher level of moral disengagement toward refugees (Kirişçi and Güner 2024).

These findings can be associated not only with the social change theory but also with the *cognitive dissonance theory*. This theory, developed by Festinger (1957), argues that when individuals experience incongruence between their beliefs, values, and behaviors, they feel psychological discomfort and tend to change their behaviors or beliefs to reduce this discomfort. The fact that social influence leads individuals to deviate from their moral values towards refugees can be seen as a result of individuals' efforts to resolve this dissonance. Under environmental pressures, individuals may try to resolve this dissonance by abandoning or redefining their ethical norms (Harmon‐Jones and Mills 1999).

In addition, *social norms theory* is also in line with these findings. Social norms theory argues that individuals' behaviors are largely shaped by perceived norms (Cialdini et al. 1990). Our research has shown that individuals tend to develop moral detachment towards refugees as a result of normative pressures from their social environment. This finding emphasizes the influence of social norms on individuals' ethical decision‐making processes. In particular, external social norms may cause individuals to deviate from their moral values towards refugees, which makes an important contribution to understanding how social norms shape individuals' moral judgments.

In addition, *social identity theory* can also be evaluated in this context. Tajfel and Turner (1979) argued that individuals evaluate the social world through the group identities to which they belong and that these identities can affect individuals' moral attitudes. In our research, it is seen that the effect of social influence on moral disengagement may stem from individuals' perceptions based on their group identities. In particular, attitudes towards individuals perceived as “out‐group” such as refugees may increase individuals' tendency to develop moral disengagement. These findings make an important contribution to understanding the impact of group identities on social norms and ethical values. In addition, *symbolic interactionism theory* argues that individuals shape their norms and values as a result of their interactions with their social environment (Mead 1934). The findings of the study showed that individuals experienced changes in their moral values towards refugees under environmental pressures and social norms. This emphasizes the importance of symbolic interactionism theory in understanding the moral disengagement processes of individuals.

The critical role of moral ambivalence in individuals' moral processes is in strong agreement with the previous literature and our research contributes to expanding the literature on this topic. Moral ambivalence refers to the incompatibility between individuals' internal values and beliefs and external social norms and expectations. This can have a profound impact on individuals' moral attitudes and decision‐making processes (Hill and Pargament 2003; Pargament et al. 2011; Ekşi and Okan 2021). For example, Hill and Pargament (2003) discussed in detail how individuals' spiritual contradictions affect their psychological and moral processes and suggested that these contradictions may weaken individuals' mental health and commitment to ethical norms.

Our finding that moral ambivalence functions as a mechanism mediating the effect of social influence on moral disengagement toward refugees supports this literature. Spiritual contradictions cause individuals to experience a contradiction between the pressures from their social environment and their own inner beliefs, which paves the way for them to deviate from ethical norms (Exline et al. 2000; Ano and Vasconcelles 2005; Şahin and Okan 2024; Okan and Şahin 2024). These findings are particularly important in understanding the impact of moral contradictions on attitudes towards “marginalized” groups such as refugees. The tension between social norms and individuals' religious or spiritual values may contribute to an increase in moral disengagement.

In our study, spiritual ambivalence was found to partially mediate the effect of social influence on moral disengagement. This suggests that individuals' coping with spiritual contradictions may play a critical role in maintaining their moral consistency. Pargament et al.’s (2011) studies on spiritual coping strategies emphasize how individuals' methods of coping with spiritual contradictions can affect their ethical decision‐making processes. In this context, it may be possible for individuals to reduce moral disengagement by restructuring their spiritual values or developing resistance to social pressures. Similarly, Exline et al. (2000) argued that spiritual contradictions may lead to not only psychological stress but also moral deviations in individuals. Our research further advances the literature in this area by demonstrating that spiritual contradiction functions as a mechanism that reinforces the link between social influence and moral disengagement. These findings suggest that the development of strategies that support individuals in coping with spiritual contradiction can be used to improve moral attitudes toward refugees.

Willpower is a critical factor that determines the capacity of individuals to resist social and moral pressures. In our research, it was clearly demonstrated that willpower plays a role in limiting the negative effects of social influences on moral detachment towards refugees. Willpower refers to the self‐regulation capacity of individuals and plays a decisive role in both psychological and social processes (Baumeister et al. 2007). The long‐term stress study conducted by the American Psychological Association (APA) in 2012 revealed that lack of willpower lies at the root of many problems experienced by individuals. Studies on willpower have shown that this capacity is not only limited to the ability of individuals to control themselves but also affects the ability to resist social norms and pressures. Baumeister et al. (1998) emphasized that willpower supports individuals in achieving their long‐term goals and making ethical decisions. Tangney et al. (2004), on the other hand, found that individuals with low levels of willpower are more open to social pressures and this may facilitate moral disengagement processes. In this context, our study makes an important contribution to the literature by demonstrating that willpower provides a mechanism to counterbalance the negative effects of social influences on moral disengagement towards refugees.

Will has a significant impact not only at the individual level but also in societal and social contexts. Haggard (2008) stated that seeing unconscious cerebral activities as responsible for our conscious behavior may eliminate the concept of free will and this may cause great ethical confusion. Therefore, understanding the willpower of individuals and how they can use this power against social pressures has been a critical research topic in social psychology and ethics.

The findings of this study must be interpreted within the specific cultural context in which they were obtained. In contrast to numerous Western studies, which frequently explore moral disengagement and social influence in individualistic societies, this research was conducted in a culturally collectivist context, where social norms, communal values, and group conformity play a more significant role in shaping attitudes and behaviors (Hofstede 2001). In collectivist societies, moral decision‐making is frequently deeply embedded in social expectations, rendering individuals more susceptible to social influence when forming attitudes toward outgroup members, such as refugees (Triandis 1995). However, the findings of this study demonstrate that willpower can function as a protective mechanism even in highly collectivist cultures, thereby enabling individuals to resist social pressure and moral disengagement. This finding stands in contrast to the findings of Western‐based research, which tends to emphasize individual moral agency over group‐driven influences (Aquino et al. 2009; Bandura 1999). By highlighting the cultural dynamics of moral disengagement and social influence, this study offers a more nuanced understanding of how individuals navigate moral contradictions in societies where ingroup loyalty and social harmony are highly valued. Future research should continue to investigate cultural moderators in moral disengagement processes to better capture the diverse ways in which morality is shaped by societal structures.

The present study found that individuals with strong willpower were more resistant to social pressures, and their levels of moral disengagement towards refugees were lower. This suggests that willpower serves as a fundamental self‐regulatory mechanism, allowing individuals to resist the negative consequences of social influence. These findings align with existing research in social psychology, which emphasizes the role of self‐control in moderating conformity to group norms (Baumeister et al. 2007). Prior studies indicate that moral disengagement is often facilitated by social identification with in‐groups, leading to biased moral justifications for exclusionary attitudes (Bandura 1999; Haslam 2006). However, the present findings suggest that individuals with higher willpower are less susceptible to such ingroup biases, likely because they engage in more deliberate moral reasoning, thereby resisting group‐based moral disengagement (Aquino et al. 2009). From a group dynamics perspective, research has shown that social norms shape intergroup attitudes, particularly in contexts of intergroup conflict and discrimination (Tajfel and Turner 2004). However, willpower may function as a protective factor, buffering individuals from adopting dominant discriminatory social norms and fostering a more principled moral stance. This suggests that policies and social integration strategies for refugees should not only focus on structural adjustments but also incorporate psychological interventions aimed at strengthening individuals' capacity to exercise willpower. In line with studies on moral self‐regulation (Hardy and Carlo 2011), the findings of this study highlight that enhancing self‐regulatory skills may help mitigate the tendency to morally disengage in intergroup contexts. This lends further support to the notion that willpower fulfills a pivotal regulatory function in upholding ethical conduct, particularly in contexts where social pressures might otherwise engender a disregard for moral principles.

### 4.3 Implications for Applications

The findings of this study highlight the importance of taking social psychological processes into account in educational systems, social policies, and integration programs, especially for immigrants. The complex dynamics between social influence, moral contradiction, and willpower were found to deeply influence individuals' moral processes and ethical attitudes. In this context, the following recommendations can be put forward:

**Social Integration Programs for Migrants**

Innovative strategies should be developed to support migrants' spiritual contradictions and willpower. These programs should include spiritual support mechanisms to reduce the pressure on migrants to adapt to social norms. For example, group work or psychological counseling services can be offered to develop skills to cope with spiritual contradictions. In addition, the capacity of individuals to develop resistance against social pressures can be increased through will empowerment training.

**Applications in Education System**

Social psychological approaches that will enable students to develop resistance against social pressures should be adopted. In this context, curriculum arrangements can be made to develop critical thinking skills. Studies that support students' ethical decision‐making processes may help them to be less affected by the damaging aspects of social influences. For example, simulations to understand group dynamics, practices that develop empathy, and ethical discussion activities can be among such approaches.

**Inter‐Institutional Cooperation**

Strong cooperation between educational institutions, social service providers, and government policies should be ensured when developing strategies for the integration of migrants and for individuals to cope with social Influences. In this context, collective efforts to reduce the risk of moral disengagement should be encouraged.

**Theoretical Contributions**

The research has brought an innovative model to the field of social psychology. It has made an important contribution to the theoretical framework by revealing the effects of social influence, moral contradiction, and willpower on moral processes. This model offers a new perspective in understanding the impact of social psychological factors on individuals' ethical attitudes.

### 4.4 Limitations and Future Studies

#### 4.4.1 Limitations

This study was conducted with a sample of university students only. This limits the generalizability of the results to different age groups, occupations, or cultural contexts. The study utilized a cross‐sectional design, which created limitations in understanding the dynamics of the relationships between variables over time. In particular, there is a need for longitudinal research that examines how the influence of social influences and moral contradictions on individuals' moral processes changes over time.

#### 4.4.2 Recommendations

##### Different Demographic Groups

Replication of this study in different age groups, occupations, and cultural contexts is critical to increase the generalizability of the findings.

##### Longitudinal Designs

Since this study is based on a cross‐sectional design, it cannot fully explain the dynamics of the relationships between variables over time. Therefore, the following issues can be examined by using longitudinal designs:

##### Experimental Research

Experimental designs can be used to understand how moral ambivalence and willpower influence the decision‐making processes of individuals under social influence. For example:

##### Psychological Interventions

The findings of this study may guide the development of psychological interventions to increase individuals' ability to cope with spiritual contradictions. In this context:

##### Use of Technological Tools

Technological tools can be used to examine the impact of social influences on individuals' moral processes in wider audiences. For example, online surveys, mobile applications, or social media platforms can enable real‐time monitoring of social pressure and moral processes.

## Author Contributions

**Nesrullah Okan**: conceptualization, investigation, funding acquisition, writing – original draft, methodology, validation, visualization, writing – review and editing, software, formal analysis, project administration, data curation, supervision, resources.

## Conflicts of Interest

The author declares no conflicts of interest.

### Peer Review

The peer review history for this article is available at https://publons.com/publon/10.1002/brb3.70506.

## Data Availability

The datasets generated during and/or analyzed during the current study are available from the corresponding author on reasonable request.
